# Thermostructural Numerical Analysis of the Thrust Chamber of a Liquid Propellant Rocket Engine

**DOI:** 10.3390/ma15155427

**Published:** 2022-08-07

**Authors:** Roberto Citarella, Michele Ferraiuolo, Michele Perrella, Venanzio Giannella

**Affiliations:** 1 Department of Industrial Engineering, University of Salerno, 84084 Fisciano, SA, Italy; 2 Italian Aerospace Research Center (CIRA), Thermostructures and Thermal Control Technologies and Design Lab., Via Maiorise Snc, 81043 Capua, CE, Italy; 3 Department of Chemical, Materials and Production Engineering, University of Naples Federico II, 80125 Naples, NA, Italy

**Keywords:** liquid rocket engine, finite element method, stress analysis, thermal stress, plasticity, creep, low-cycle fatigue

## Abstract

The numerical simulation of rocket engine thrust chambers is very challenging as several damaging phenomena, such as plasticity, low-cycle-fatigue (LCF) and creep occur during its service life. The possibility of simulating the thermostructural behavior of the engine, by means of non-linear finite element analyses, allows the engineers to guarantee the structural safety of the structure. This document reports the numerical simulations developed with the aim of predicting the thermostructural behaviour and the service life of the thrust chamber of a liquid-propellant rocket engine. The work represents a step ahead of previous researches by the authors, with particular reference to the addition of the Smith-Watson-Topper (SWT) fatigue criterion, and to the implementation of a sub-modelling technique, for a more accurate assessment of the most critical section of the component. It was found that the equivalent plastic strains in the most critical nodes obtained through the sub-modelling technique were about 20% lower than those calculated without sub-modelling. Consistently with experimental tests from literature conducted on similar geometries, the most critical areas resulted to be on the internal surface of the chamber. The analyses demonstrated that the LCF damaging contribution was significant, with a life prediction for the thrust chamber of about 3400 cycles.

## 1. Introduction

The investigation presented in this work was conducted within the frame of the HYPROB Program, a research project aimed at developing and implementing technologies for regeneratively-cooled liquid-propellant rocket engines, which use liquid methane and liquid oxygen. Since elevated thrust values are requested, rather high chamber pressures and heavy heat fluxes are predicted in the combustion chamber. Consequently, active cooling solutions are needed to maintain the adopted materials at suitably low temperatures, i.e., 500–800 K.

From the thermo-mechanical point of view, the pressure inside the cooling channel must be higher than the pressure of the combustion gases in the thrust chamber causing, as a result, bending of the inner wall separating the hot gases from the coolant flow; furthermore, compressive hoop stresses arise in the inner wall since its thermal expansion is restrained by the external cold structure. The induced thermal stresses generally exceed the yield stress generating significant plastic deformations in the hot-gas side walls. The failure mechanisms of the thrust chamber have been investigated in several experimental campaigns. For instance, Hannum et al. [1] and Quentmeyer [2] investigated the mechanical behavior of the cooling channel conducting several tests on subscale demonstrators. More recently, Gernoth et al. [3] conducted several thermo-mechanical fatigue tests on simplified geometries with realistic boundary conditions useful for the validation of the structural analyses of thermally loaded structures. Then, several efforts have also been made to implement accurate viscoplastic models able to reproduce the actual deformation of the cooling channel [4,5]. The present paper is a continuation and improvement of a previous work [6] by the authors, whose aim was to analyze, from a numerical point of view, the main causes of failure of a liquid rocket engine thrust chamber.

The thrust chamber structure is composed by electrodeposited layers made of copper and nickel on the outside with a CuCrZr alloy element on the inside. A regenerative cooling system was adopted to remove heat from the inner structure of the thrust chamber, through a flux of methane in the axial cooling channels. Being a regenerative cooling system, the same cooling fluid is also injected as fuel into the combustion chamber. Elevated thermal gradients exist through the inner chamber wall (ligament) that separates the cooling channels from the hot gas. In this context, several efforts have been made to optimize the regenerative cooling channel. Xu et al. [7] established a design model based on the back propagation neural network to improve the overall cooling performances. Atefi et al., presented an optimization of channel dimensions by minimizing the thermal resistance between cooling fluid and hot gas with a defined pressure drop [8].

Furthermore, several efforts have been made to study the thermal efficiency of the cooling system of regeneratively cooled thrust chambers by means of thermal and fluid-dynamic numerical investigations [9,10].

In general, the thermomechanical behavior of thrust chambers of liquid rocket engines is described by means of a model allowing for combined hardening (isotropic and kinematic hardening) and creep. Several works demonstrated that the best suited kinematic hardening models are the nonlinear ones, based on Armstrong and Frederick [11,12,13,14,15,16] or Chaboche [11] approaches (the latter resulting from the superposition of two or more Armstrong and Frederick models). Several authors have adopted at least three nonlinear hardening models in order to properly simulate the ratcheting behavior, i.e., the progressive plastic strain accumulation during cycling [12]. On the other hand, creep phenomena are usually modelled in literature with the Norton’s law with allowance for just secondary creep [12], whereas a more accurate modelling was preferred in this work accounting for both primary and secondary creep stages, obtained by means of the combined time hardening rule [17,18].

Regarding the investigation of typical thrust chamber failure mechanisms, creep, Low-Cycle Fatigue (LCF) and ratcheting have been identified as the most likely causes of failure [19,20,21,22,23]. Typically, a “dog-house” effect is recorded between the cooling channels as a result of a combination of those failure mechanisms. Then, several researchers have tried to implement a damage model to account for these phenomena [24], and to derive a reliable prediction of the thrust chamber service life. Dresia et al. trained an artificial neural network to predict the fatigue life of liquid rocket engine combustion chambers combining low cycle fatigue with ductile failure caused by ratcheting effects during post processing [25]. Previous works adopted the Manson-Coffin-law for LCF estimations [15,16]. However, no studies can be found on the investigation of the effects of multiaxial and non-proportional loading on the fatigue life of the thrust chamber. Hence, a significant effort was made to fill this gap in the present study, so as to gain a better understanding of how important these effects could be.

Since the experimental testing activity for these applications can be very expensive, it is mandatory to minimize experiments by adopting high fidelity numerical models. Within this context, the accuracy of the numerical modelling obtained in a previous investigation by the authors [6] was improved thanks to the current investigation. Particularly, the main advances with respect to the aforementioned work were summarized here:

- A time step sensitivity analyses was conducted in order to better describe those phases where very steep temperature changes occur in very short time periods, i.e., during ignition and purging phases.
- A sub-modelling approach was implemented to transfer boundary conditions from a global coarse model to a local fine model (in [6], simplified boundary conditions were applied to the cut boundaries of the local model with no assessments for the global model).
- A relaxation phase was added after the purging phase in the thermomechanical loading cycle: during this phase, the thrust chamber slowly returns to environmental thermal conditions, with consequent relaxation of thermal stresses.
- A sensitivity study on the influence of the global model analyses on the results of the local model was carried out.
- LCF evaluations method were conducted by means of the Smith-Watson and Topper (SWT) criterion using the commercial code *ncode DesignLife*.

The rest of this paper can be summarized as it follows. Section 2 reports the adopted mathematical models for both thermal and structural analyses with the related governing equations. Section 3 describes the numerical FEM models. Section 4 defines the thermomechanical loading conditions. Finally, time step convergence studies and sensitivity analyses on the impact of the global model analyses were presented and discussed in Section 5 and Section 6, respectively. Final results and the concluding remarks were reported in Section 7 and Section 8.

## 2. Structural Model

**Figure 1:**
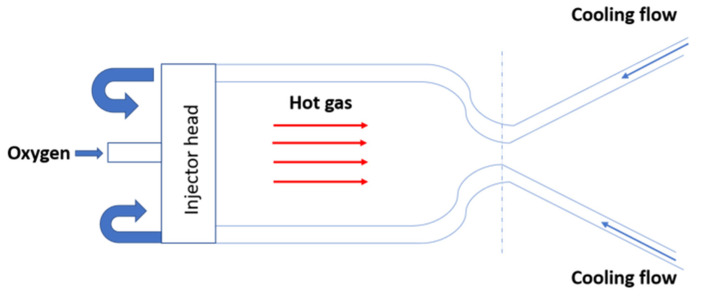
Rocket engine thrust chamber and related functional scheme.

**Figure 2:**
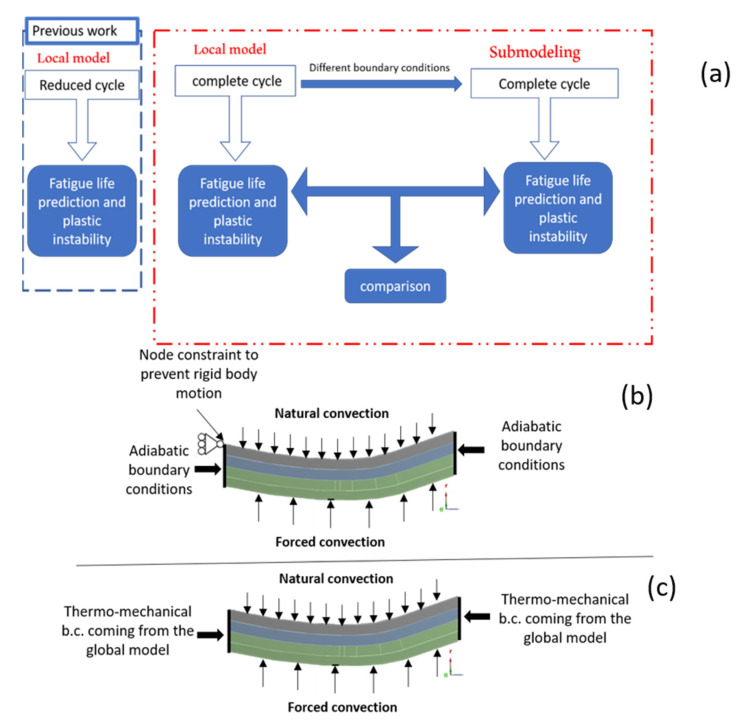
(**a**) Schematic representation of the modelling strategy; (**b**) local model boundary conditions; (**c**) sub-modelling boundary conditions.

A schematic of the rocket engine thrust chamber is reported in Figure 1. Figure 2a shows a schematic view of the adopted numerical modelling strategy, highlighting the above-mentioned improvements provided by the present work. The boundary conditions applied in the previous work and those considered with the proposed sub-modelling approach are summarized in Figure 2b,c.

In this paper, plasticity was modelled by the von Mises yield criterion, Prandtl-Reuss flow rule, and both isotropic and non-linear Chaboche kinematic hardening laws. The combined time hardening creep model was selected to simulate primary and secondary creep effects [20]. A cumulative damage model, considering the effects of plastic instability (thermal ratcheting), fatigue and creep, was used in order to predict the cycles number to failure.

A one-way thermal-structural coupling was employed. Hence, the temperature field influences the strain/stress response, and not vice versa. Several papers from literature demonstrated that such an approximation provided accurate results [26,27,28,29].

In this work a FEM-FEM submodelling approach was adopted but alternative schemes would be also possible, e.g., FEM-DBEM approaches [30,31].

The transient thermal behaviour of cooling channels was investigated by non linear FEM analyses, considering the dependence of material properties with temperature. More information about these aspects can be found in [6,32].

### 2.1. Plasticity and Creep

**Figure 3:**
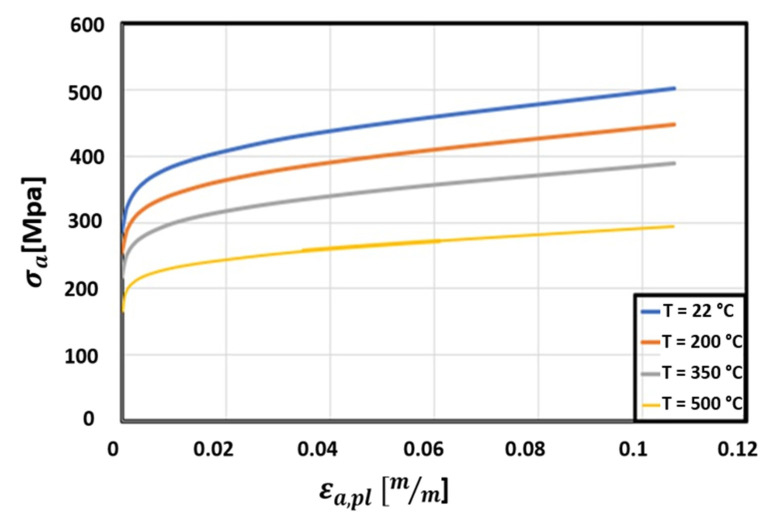
Stress-strain curves for CuCrZr at different temperature levels [18].

Inelastic behavior of the thrust chamber was modeled assuming a combined isotropic-kinematic hardening model; in particular, a multilinear isotropic hardening model and a nonlinear Chaboche equation were considered, whilst creep effect was modeled by a combined time hardening relationship; the corresponding parameters are summarized in Table 1. A more extensive discussion on the adopted viscoplastic model and a comparison among different approaches are described in [20]. The parameters of multilinear isotropic model resulted by extrapolating data from experimental outcomes in terms of stabilized stress-plastic strain curves at different temperatures, available for CuCrZr material [19]. The Chaboche kinematic hardening law with three back stresses, according to the Armstrong–Frederick nonlinear model, was employed to include the kinematic hardening effects (see Figure 3) [32,33,34]. The combined time hardening model can be described through the following relationship:

(1) $$\varepsilon_{cr}=\frac{D_{1}\sigma^{D_{2}}t^{D_{3}+1}e^{-\frac{D_{4}}{T}}}{D_{3}+1}+D_{5}\sigma^{D_{6}}te^{-\frac{D_{7}}{T}}$$

Further details of the creep and plasticity hardening models adopted can be found in [6].

### 2.2. Low Cycle Fatigue

The Smith-Watson-Topper life equation always accounts for mean stress and is typically used for low cycle fatigue; it defines a damage parameter based on the product of the maximum stress of each cycle $\sigma_{max}$ and the strain amplitude $\varepsilon_{a}$. Then, the following equation is solved for life/damage assessment:

(2) $$\varepsilon_{a}\sigma_{max}=\frac{\sigma_{f}^{\prime }}{E}\;{\left(2N_{f}\right)}^{b}+\varepsilon_{f}^{\prime }{\left(2N_{f}\right)}^{c}$$

where the exponent b and the fatigue strength coefficient $\sigma_{f}^{\prime }$ are coefficients of the Basquin law, whilst the exponent c and the fatigue ductility coefficient $\varepsilon_{f}^{\prime }$ are parameters of the Manson-Coffin curve and *2N_f_* is the number of reversals to failure. Multiaxiality effects are also considered and a biaxiality ratio $B_{r}$ (ratio between the second principal stress and the first principal stress) is evaluated:

(3) $$B_{r}=\frac{\sigma_{2}}{\sigma_{1}}$$

## 3. Description of Numerical Model

**Figure 4:**
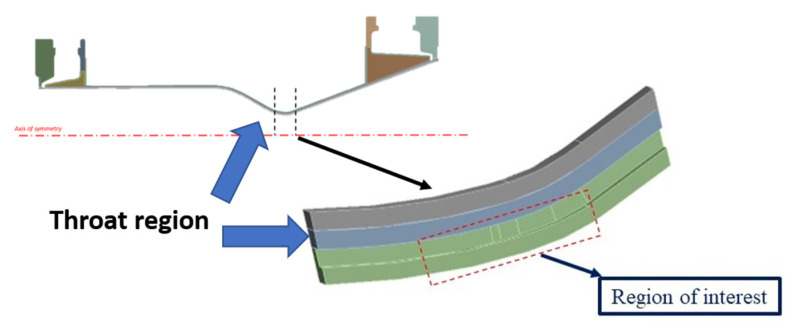
Regions of interest numerically analyzed.

Because the maximum values of heat flux are expected in the throat region of the combustion chamber (see Figure 4), a local model of the throat region was analyzed to reduce the computational burden required to investigate the whole chamber, see Figure 4. A numerical analysis of the global model with the same element size of the local model was also conducted; the saved computation time is significant as the global model analysis is about 12 times slower than that detected adopting the sub-modelling approach.

As mentioned earlier, instead of assigning predefined boundary conditions on the cut surfaces of the throat area (as conducted in [6]), a sub-modeling approach was adopted. Namely, a global coarse model was built to accurately evaluate the displacement and thermal boundary conditions to be applied on the cut surfaces of the local model [32].

In general, sub-modelling approaches allow to restrict the mesh refinement to the zones where plastic strains are envisaged. In particular, FEM analyses on a global model with a coarse mesh and a local model with a fine mesh are sequentially conducted. The displacements on the interface between the global and local models, obtained by a FEM linear analysis of the global model, are transferred to the local nonlinear model as boundary conditions for each time step. In the current work, the number of cycles simulated in the global model were less than those simulated in the local model in order to save computation time, namely at a certain point the results of the global model were considered stabilized and identically applied to all the subsequent further simulated cycles on the local model. In addition, a sensitivity analysis on the required number of loading cycles to be simulated in the global model was conducted in order to minimize the computation time (see next Section).

**Figure 5:**
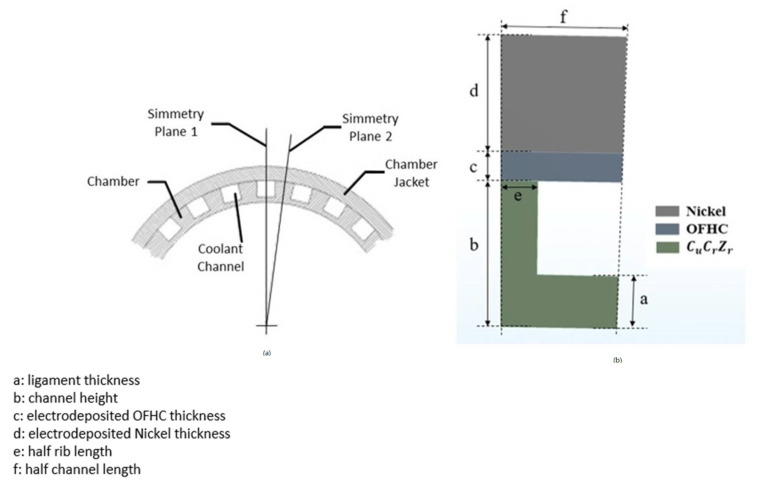
Highlight of symmetry conditions (**a**) and cooling channel section (**b**).

**Figure 6:**
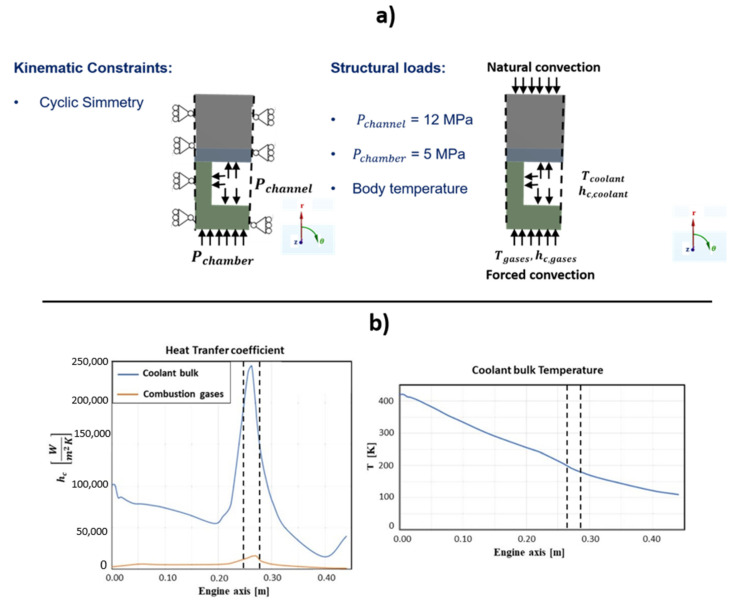
(**a**) Thermal and structural boundary conditions; (**b**) Convective boundary conditions.

By considering the symmetry of structure, only a half-cooling channel was modeled (Figure 5) with related boundary conditions (Figure 6).

The combustion chamber was realized with three different materials, as shown in Figure 5:

- CuCrZr alloy, in the zone in contact with the hot gases and the coolant;
- a thin layer of electrodeposited oxygen-free high-thermal conductivity copper (OFHC Cu);
- a layer of electrodeposited nickel to afford adequate chamber stiffness.

Table 2, Table 3, Table 4, Table 5, Table 6 and Table 7 report the mechanical, physical and thermal properties of the copper alloy CuCrZr, the electrodeposited OFHC Cu material, and nickel [6].

Pressures of the hot gases and of the cooling fluid were considered as uniform and equal to 5 and 12 MPa, respectively. The thermal and structural boundary conditions are shown in Figure 6. Coolant bulk temperatures, combustion gases and their respective heat transfer coefficients, which varied along the axis of chamber, were carried out by computational fluid dynamics (CFD) analyses [35,36]. The heat transfer coefficients and bulk temperatures to be applied in the local model thermal analyses, ranged between the dashed lines in Figure 6b. Natural convection conditions were considered on the external surface of the closeout structure. Displacement and temperature boundary conditions applied to the cut surfaces were retrieved by the results of thermal and structural analyses performed on the global model. Finally, the initial temperature was set to 293 K.

With regard to the purging and relaxation phases the heat transfer coefficients and bulk temperatures considered are summarized in Table 8. The values were retrieved from literature [12].

**Figure 7:**
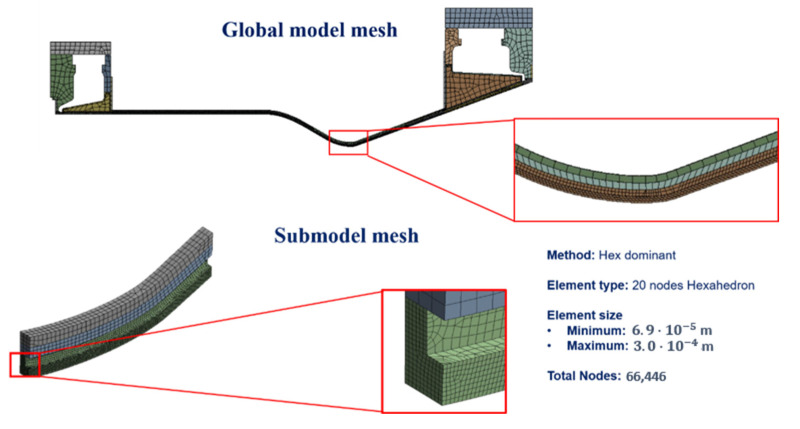
Adopted FEM mesh adopted, as obtained from convergence analysis.

The finite element model used for the thermal and structural analyses, chosen after conducting a grid convergence analysis in the previous work [6], is depicted in Figure 7. The results of the convergence analysis are reported in Table 9. The node at which thermal and structural results are retrieved lies on the internal surface of the chamber, where maximum plastic strains are detected.

## 4. Thermostructural Cycle Description

Four sequential phases were considered to define each thermal–structural loading:

- an ignition transient phase with duration of 3 s, in which the activation of thermal loads occurs;
- a hot phase with duration of 100 s,
- a purging phase of 3 s duration, during which an injection of liquid oxygen is performed to eliminate any waste into the channels;
- a relaxation phase, lasting almost 4000 s, when only natural convection acts to recover the chamber temperature back to room temperature.

Creep is activated in the ignition and hot phases where maximum temperature values are expected (this provided a further refinement in comparison to [6] where creep conditions where only activated during the hot phase).

**Figure 8:**
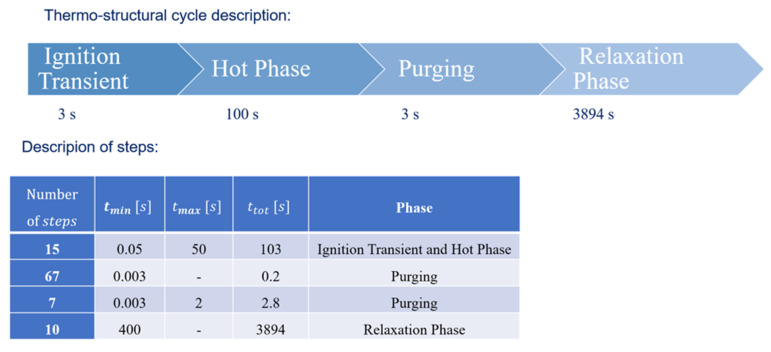
Thermo-structural cycle description.

In Figure 8 the phases composing the simulated thermo-mechanical load cycle are reported. Appropriate time step sizes were derived by means of a convergence analysis.

There is no combustion in thrust chamber during the purging and relaxation phases and, so, heat convective transfer coefficients and pressures were considered as null. When the combustion occurs, i.e., during the ignition and hot phase, chamber pressures and convective heat fluxes are generated on the inner surface.

The life predictions model is the same as adopted in [6]. In summary, a total usage factor is evaluated summing the effects of creep, fatigue and ratcheting.

## 5. Time Step Convergence

A time step convergence analysis was conducted since the choice of the time step size could have a significant impact on the final results. Especially in the ignition and in the purging phase, temperature variations over time were very steep, with the consequent need to set very small time steps, especially at the beginning of those phases. In particular, an automatic time step algorithm was selected, choosing the initial, minimum and maximum time step sizes; these were automatically increased or decreased during the progression of analysis. More specifically, in the time stepping algorithm used by ANSYS the time increment at the time step *n* + 1, $∆t_{n+1}$, is considered to range between the following values:

(4) $$\max\left(\frac{∆t_{n}}{2},∆t_{min}\right)<∆t_{n+1}<min\left(2∆t_{n},\;∆t_{max}\right)$$

**Figure 9:**
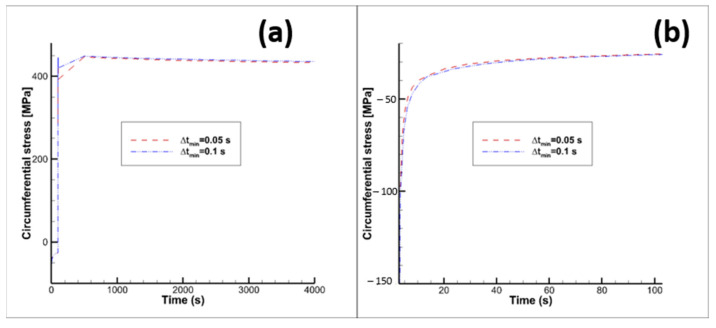
Circumferential stress vs time. (**a**) overall 1st cycle, (**b**) close up of hot phase 1st cycle. Orange curve (“previous”): $∆t_{min}=0.1\;s$ for the hot phase and $∆t_{min}=0.006\;s\;$ for the purging phase; blue curve (“new”): $∆t_{min}=0.05\;s$ for the hot phase and $∆t_{min}=0.003\;s$ for the purging phase.

Figure 9 shows the results obtained picking $∆t_{min}=0.1\;s$ for the hot phase and $∆t_{min}=0.006\;s$ for the purging phase (orange curve), and $∆t_{min}$= 0.05 s for the hot phase and $∆t_{min}$= 0.003 s for the purging phase (blue curve).

## 6. Influence of Global Model Analyses

A sensitivity study of the impact of the number of loading cycles, simulated on the global model, on the equivalent plastic strain outcome was performed. In fact, in order to save computation time, it is preferable to minimize the number of cycles simulated in the global model and apply the displacement boundary conditions of the last global model cycle for all the subsequent loading cycles analyzed in the local model. Hence, prior to establishing the number of cycles to simulate in the global model, maximum and minimum total displacements on the cut boundaries were evaluated with 10 global model loading cycles; then, the maximum percentage difference values between the 10th and the *i*th cycles were estimated in order to understand how different are the displacement boundary conditions if the number of loading cycles simulated in the global model are less than 10. The maximum percentage difference MPD is evaluated as follows:

(5) $$MPD_{i}=\frac{\left|\max\left(s_{i}\left(\bar{t}\right)-s_{10}\left(\bar{t}\right)\right)\right|}{\left|s_{10}\left(\bar{t}\right)\right|}\cdot 100\;\;\;\forall \;\;0\leq \bar{t}\leq 4000\;$$

where:

- $\bar{t}$ is a time instant of the complete loading cycle, which lasts 4000 s;
- $s_{i}\left(\bar{t}\right)$ is the displacement magnitude evaluated at the time instant $\bar{t}$ for the *i*th loading cycle;
- $s_{10}\left(\bar{t}\right)$ is the displacement magnitude evaluated at the time instant $\bar{t}$ for the 10th loading cycle.

**Figure 10:**
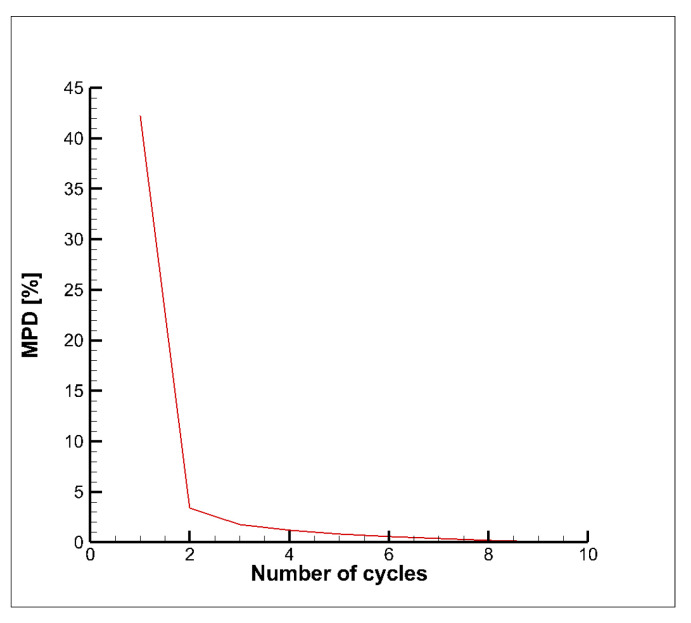
Maximum percentage difference MPD.

Three cases were considered for comparison, considering the simulation of 10, 5 and 3 global model loading cycles: the $MPD_{i}$ values for those cases are all smaller than 4% (see Figure 10).

**Figure 11:**
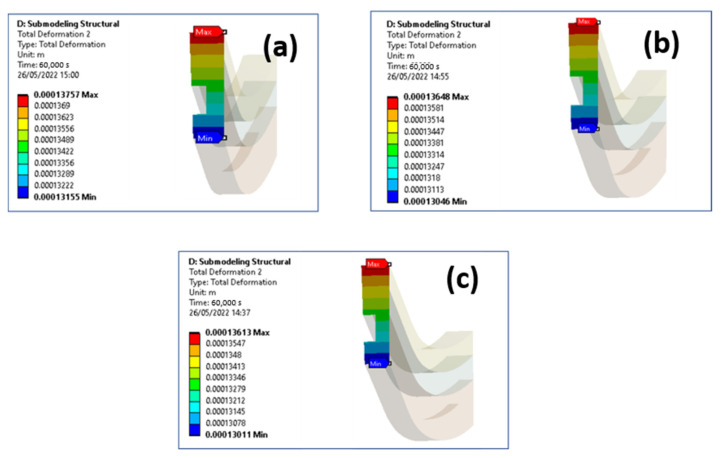
Total displacement contour plot (end of 15th cycle) at a cut boundary considering: (**a**) 3 global model cycles, (**b**) 5 global model cycles, (**c**) 10 global model cycles.

Figure 11 shows how similar are the displacement magnitude contour plots at the end of the 15th cycle considering the following numbers of simulated global model loading cycles: (a) 3, (b) 5 and (c) 10.

Then, the results of this study demonstrated that it is convenient to simulate only 3 loading cycles in the global model; indeed, the maximum percentage difference in terms of equivalent plastic strain (in the node where maximum plastic strains were detected), with respect to the case where 10 global model loading cycles were simulated, resulted of only 1.1%. In such a way a 23% run time saving on the global model analyses was obtained.

## 7. Results and Discussion

**Figure 12:**
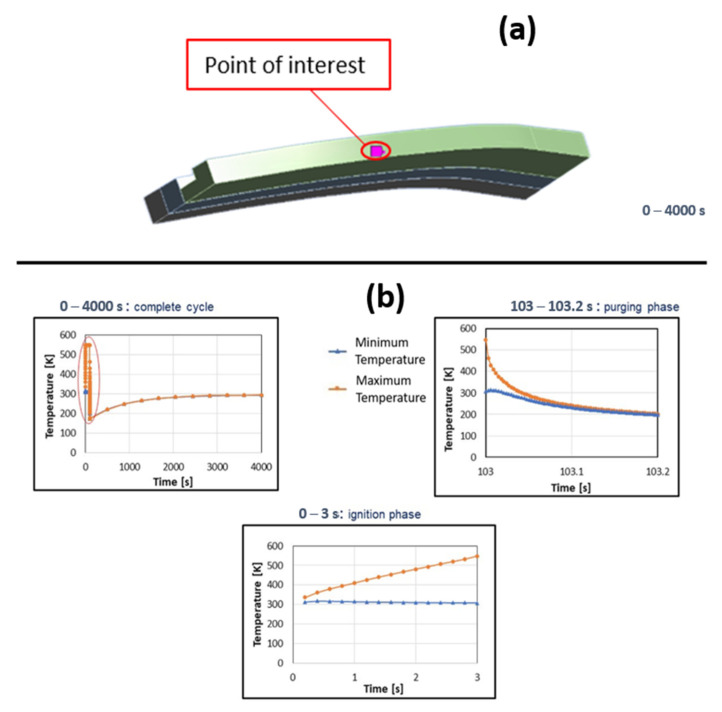
(**a**) Location of the node with the maximum equivalent plastic strain value, (**b**) Maximum and minimum temperature variations over time at the point of interest.

**Figure 13:**
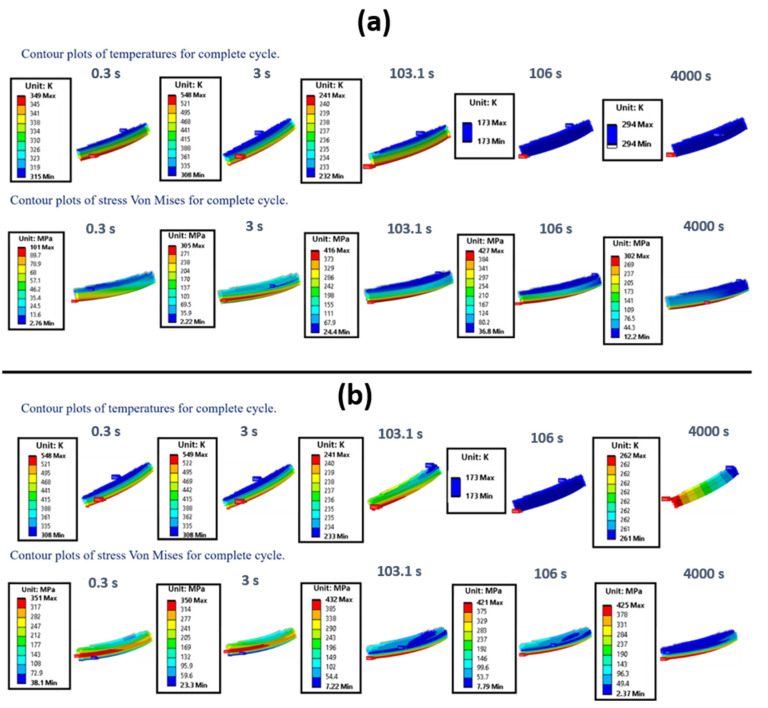
Temperature and von Mises stress contour plots at the most significant time instants of the first cycle, considering predefined boundary conditions on the cut surfaces (**a**), and the displacement/temperature on the cut surfaces evaluated by FEM global analysis (**b**).

**Figure 14:**
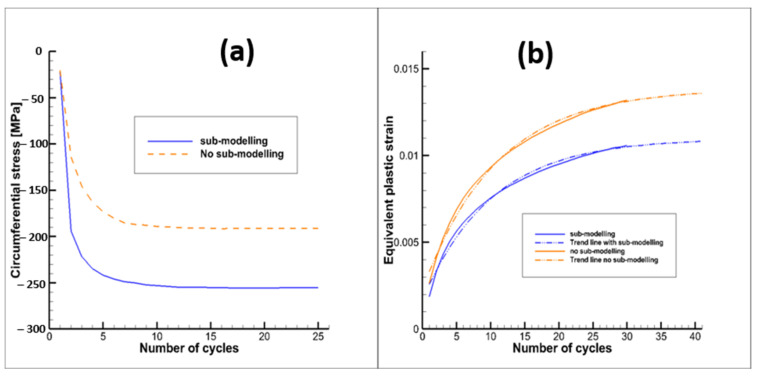
(**a**) Circumferential stress vs time at the end of the hot phase, (**b**) Equivalent plastic strain evolution with number of cycles.

The main outcomes of the thermal–mechanical analyses carried out on the throat region are summarized here. Figure 12a shows the location of the node where the maximum equivalent plastic strains were detected; in the following, temperature, strains and stresses are referred to that node. Maximum and minimum temperature variations over time are depicted in Figure 12b: it is worth highlighting that during the purging phase temperatures change very quickly with the consequent impact on plastic strains in the ligament (as detailed in the following). Temperature and von Mises stress contour plots are compared at selected time instants of the first loading cycle, considering boundary conditions on cut surfaces of the local model arbitrarily predefined (Figure 13a) rather than obtained by sub-modelling approach (Figure 13b). It is easy to notice that there are no significant differences in terms of temperature and thermal gradients; on the other hand, there is a noticeable difference with regard to equivalent von Mises stresses, as due to different displacement boundary conditions. Then, as expected, also plastic and creep strains are different. As a matter of fact, the local model displacement boundary conditions, evaluated by the submodeling approach are such that compressive stresses during the hot phase are higher than those envisaged in the local model with predefined boundary conditions (see Figure 14). Furthermore, in both models, the ligament compressive stresses in the tangential direction, highlighted in the hot phase, remain compressive up to the end of the creep period. In other words, temperature and stress values detected during the hot phase, are such that creep damage can be considered negligible, as demonstrated in [27], where stress-time curves are reported for temperature values higher than those encountered in the present test case. Therefore, no creep failure predictions were needed for the life assessment. Alternatively, creep failure can become dominant when higher thrust levels are required [27].

Figure 14b shows the evolution of the equivalent plastic strain cycle by cycle for both of the considered numerical analyses. It is worth noting that the adoption of sub-modelling approaches allows one to obtain smaller equivalent plastic strains at the end of the cycle with respect to the approach with predefined boundary conditions (with no applied constraints along the axial direction). The main reason of this result is that the applied compressive boundary conditions, adopting sub-modelling techniques, allows one to obtain smaller tensile plastic strains when the thermal-mechanical loads are reversed during the purging phase.

**Figure 15:**
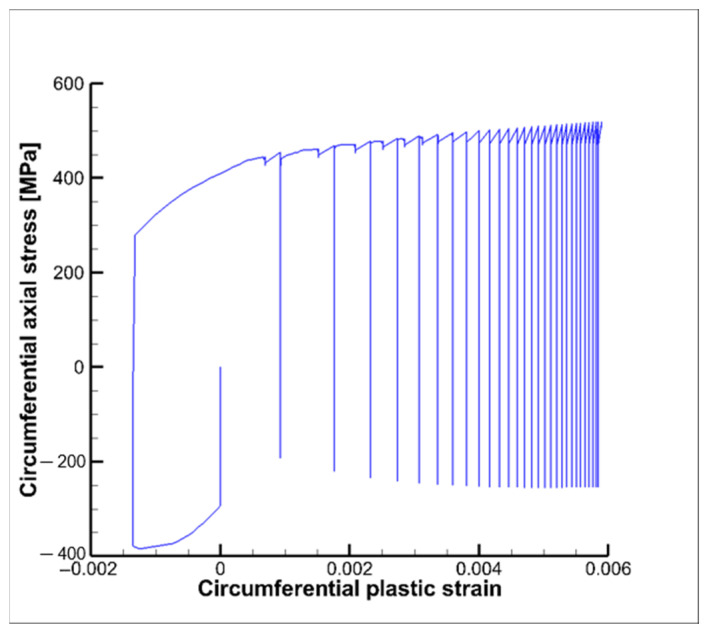
Circumferential stress vs. circumferential plastic strain.

With regard to ratcheting life prediction, the curve representing equivalent plastic strain versus number of cycles was extrapolated by means of a curve with the following expression:

(6) $$y=A+Be^{-Cx}$$

where in this case x represents the number of cycles and y the equivalent plastic deformation. As a result, equivalent plastic strains were not expected to further increase after 45–50 cycles, leading to the shakedown condition (see Figure 14b). As shakedown occurs it is clear that low cycle fatigue evaluations become useless (see Figure 15); in fact, the increment of plastic strains tends to zero as the number of cycles increases; this means that the effects of strain hardening are such that after a finite number of cycles a linear elastic behavior is expected.

**Figure 16:**
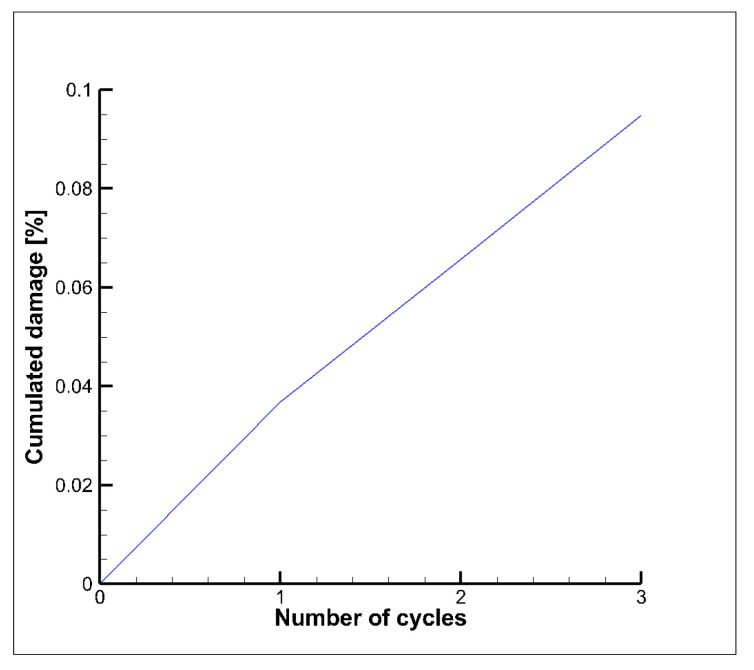
Cumulated damage vs. number of cycles—SWT method.

All the fatigue evaluations have been conducted using the commercial code *ncode DesignLife,* which works in the post-processing phase, namely after the solution of the non-linear structural analyses, and processes the calculated stress and strain field adopting the SWT method. Then, an evaluation of the cumulative fatigue damage $D_{3\;}$ for the first three cycle was carried out (see Figure 16), by means of the following relationship:

(7) $$D_{3\;}=\frac{1}{N_{1}}+\frac{1}{N_{2}}+\frac{1}{N_{3}}$$

where $N_{1}$, $N_{2}$ and $N_{3}$ represent the number of cycles to failure if all the loading cycles are equal to the first, the second or the third cycle, respectively.

The corresponding thrust chamber fatigue life $N_{f}$ (see Equation (8)), evaluated considering only the first three cycles, is equal to 3424 cycles:

(8) $$N_{f}=\frac{1}{\frac{1}{N_{1}}+\frac{1}{N_{2}}+\frac{1}{N_{3}}}$$

**Figure 17:**
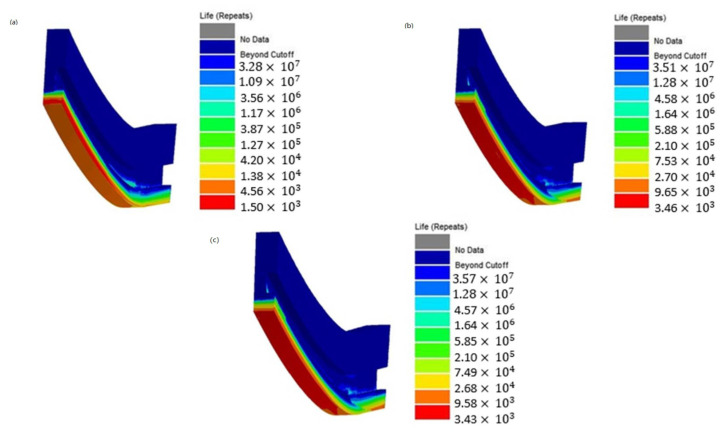
Service life: (**a**) first cycle $\left(N_{1}\right)$, (**b**) second cycle $\left(N_{2}\right)$, (**c**) third cycle $\left(N_{3}\right)$.

Since the hysteresis curves for the following loading cycles tend to shrink, as highlighted in Figure 15, the corresponding damage contribution is expected to decrease up to nearly zero values after 20–25 cycles. Figure 17 shows the service life contour plots for the first, second and third cycle. As expected, the minimum values occur in the internal surface of the cooling channel where the highest temperatures and thermal gradients are envisaged because of the heating of the hot gases.

A very small non-proportional loading factor, equal to 0.002, was also estimated (usually non-proportional loading becomes significant for a factor greater than 0.25). This result is consistent with the results obtained in [6], where it was demonstrated that the curve representing the variation in the circumferential stress vs. the axial stress (load path) is very close to a straight line that passes through the axes origin. Consequently, non-proportional loading effects can be considered negligible. The definition of the non- proportional loading factor can be found in the *ncode Design Life* theory guide [37]. On the other hand, since the biaxiality ratio $B_{r}$ is equal to 0.45, multiaxiality has significant effects on the thrust chamber service life.

## 8. Conclusions

This work presented the numerical modelling of the mechanical behavior of a liquid rocket engine thrust chamber through a global-local sub-modelling approach. The work represents an extension of a previous research activity by the authors. The sub-modelling approach was implemented so as to get an improvement on the accuracy of the stress-strain field evaluation in the critical positions, in turn allowing for a more accurate life prediction of the rocket engine.

The thermomechanical load cycle was updated through the modelling of a relaxation phase after the purging-post cooling phase, thus simulating the slow cooling of the component when reaching the room temperature before the following ignition stage, thus correctly simulating repeated ground hot firing cycles. Further significant advances with respect to the previous work were obtained, from the more realistic boundary conditions computed with the sub-modelling approach, to a time step convergence study in the ignition and purging phases to better simulate all time instants with very high thermal gradients.

The results demonstrated that the adoption of the predefined boundary conditions (no submodelling), as previously considered, slightly overestimated the chamber service life. The simulation of the relaxation phase allowed one to alleviate the thermal gradients and stresses, in turn having a beneficial effect on the fatigue life prediction. Low cycle fatigue analyses have demonstrated that multiaxiality effects are significant, whereas the loading can be assumed as proportional. Finally, both creep and ratcheting phenomena resulted to provide a negligible damage along the whole component life. Even though no experimental results are available (a campaign test is scheduled for the end of the year), the results illustrated in this work are consistent with those obtained with very similar geometries and boundary conditions [12,23]. Furthermore, hot fire tests conducted in the past on similar geometries have demonstrated that the most critical areas lie on the internal surface of the chamber leading to the characteristic “dog-house” effect in the cooling channel [19]; this is consistent with what is numerically obtained here.

The computation time requested to perform a thermostructural global-local analysis with 25 loading cycles was 16 h, using an Intel Xeon Gold processor (Intel, Santa Clara, CA, USA) with 3 GHz and a RAM of 190 GB.

## Figures and Tables

**Table 1:** Material parameters for combined time hardening equation, where temperature is expressed in K, time in s, and stress in MPa [13].

*D*_1_	*D*_2_	*D*_3_	*D*_4_	*D*_5_	*D*_6_	*D*_7_
$6.05\times 10^{10}$	3	−0.92	23,695	$2.82\times 10^{-22}$	14	23,695

**Table 2:** Thermal and physical properties of CuCrZr alloy [6].

Temperature [K]	$\mathrm{Mass}\;\mathrm{Density}\;\left[kg/m^{3}\right]$	Thermal Conductivity $\left[W/mK\right]\;$	Specific Heat [J/kgK]	Thermal Expansion Coefficient [1/K]
300	8933	320	390	$15.7\times 10^{-6}$
600	8933	290	390	$17.9\times 10^{-6}$
900	8933	255	400	$18.7\times 10^{-6}$

**Table 3:** Mechanical properties of CuCrZr alloy.

Temperature [K]	Young’s Modulus E $\left[GPa\right]$	Poisson’s Ratio *ν*	Yield Stress [MPa]	Ultimate Tensile Strength [MPa]
300	130	0.3	433.9	477.9
500	106	0.3	383.3	402.9
700	87	0.3	313	329.4
900	44	0.3	156.3	174.5

**Table 4:** Physical properties of electrodeposited OFHC Cu.

Temperature [K]	$\mathrm{Mass}\;\mathrm{Density}\;\left[kg/m^{3}\right]$	Thermal Conductivity $\left[W/mK\right]\;$	Specific Heat [J/kgK]	Thermal Expansion Coefficient [1/K]
300	8913	390	385	$17.2\times 10^{-6}$

**Table 5:** Mechanical properties of the electrodeposited OFHC Cu.

Temperature [K]	Young’s Modulus E $\left[Gpa\right]$	Poisson’s Ratio *ν*	Yield Stress [MPa]	Ultimate Tensile Strength [MPa]
28	118	0.34	68	413
294	114	0.34	60	208
533	65	0.34	50	145
755	40	0.34	38	80

**Table 6:** Physical properties of the electrodeposited nickel.

Temperature [K]	$\mathrm{Mass}\;\mathrm{Density}\;\left[kg/m^{3}\right]$	Thermal Conductivity $\left[W/mK\right]\;$	Specific Heat [J/kgK]	Thermal Expansion Coefficient [1/K]
300	8913	90	444	$12.2\times 10^{-6}$

**Table 7:** Mechanical properties of the electrodeposited nickel.

Temperature [K]	Young’s Modulus E $\left[Gpa\right]$	Poisson’s Ratio *ν*	Yield Stress [MPa]	Ultimate Tensile Strength [MPa]
28	193	0.3	344	551

**Table 8:** Input data for the thermal analysis.

	Relaxation Phase	Purging Phase
Bulk Temperature (K)	300	173
Heat transfer coefficient (W/m^2^K)	50,000	5

**Table 9:** Plastic strains, creep strains, and temperature values at the end of the hot phase.

Number of Elements in the Ligament Thickness	Equivalent Creep Strain	Equivalent Plastic Strain	Temperature [K]
4	0.004896	0.001415	556
6	0.00497	0.001541	556
8	0.00499	0.001599	556

## Data Availability

Not applicable.

## Nomenclature

*σ*	von Mises stress
*ε*	Strain
$\sigma_{f}^{\prime }$	fatigue strength coefficient
$\varepsilon_{f}^{\prime }$	fatigue ductility coefficient
b	parameter of Basquin law
*c*	parameter of Manson-Coffin law
*N* *_f_*	number of cycles to failure
*E*	Young modulus
*υ*	Poisson’s ratio
*T*	Temperature
*D*_1_, …, *D*_7_	material parameters for combined time hardening creep law
MPD_i_	maximum percentage difference
*B* *_r_*	biaxiality ratio
